# Identification and validation of a hypoxia-related prognostic signature in clear cell renal cell carcinoma patients

**DOI:** 10.1097/MD.0000000000027374

**Published:** 2021-10-01

**Authors:** Zhengtian Li, Gang Du, Rong Zhao, Wenkang Yang, Chan Li, Jun Huang, Zhenpei Wen, Hening Li, Bo Zhang

**Affiliations:** a Guangxi Medical University, Nanning, China; b Department of Bone and Joint Surgery, The First Affiliated Hospital of Guangxi Medical University, Nanning, China; c Department of Orthopedics Trauma, The Third Affiliated Hospital of Guangxi Medical University, Nanning, China.

**Keywords:** clear cell renal cell carcinoma, hypoxia, nomogram, prognosis, prognostic model

## Abstract

Increasing evidence has shown that hypoxia is closely related to the development, progression, and prognosis of clear cell renal cell carcinoma (ccRCC). Nevertheless, reliable prognostic signatures based on hypoxia have not been well-established. This study aimed to establish a hypoxia-related prognostic signature and construct an optimized nomogram for patients with ccRCC.

We accessed hallmark gene sets of hypoxia, including 200 genes, and an original RNA seq dataset of ccRCC cases with integrated clinical information obtained by mining the Cancer Genome Atlas database and the International Cancer Genome Consortium (ICGC) database. Univariate Cox regression analysis and multivariate Cox proportional hazards regression were performed to identify prognostic hub genes and further established prognostic model as well as visualized the nomogram. External validation of the optimized nomogram was performed in independent cohorts from the ICGC database.

ANKZF1, ETS1, PLAUR, SERPINE1, FBP1, and PFKP were selected as prognostic hypoxia-related hub genes, and the prognostic model effectively distinguishes high-risk and low-risk patients with ccRCC. The results of receiver operating characteristic curve, risk plots, survival analysis, and independent analysis suggested that RiskScore was a useful tool and independent predictive factor. A novel prognosis nomogram optimized via RiskScore showed its promising performance in both the Cancer Genome Atlas-ccRCC cohort and an ICGC-ccRCC cohort.

Our study reveals that the differential expressions of hypoxia-related genes are associated with the overall survival of patients with ccRCC. The prognostic model we established showed a good predictive and discerning ability in ccRCC patients. The novel nomogram optimized via RiskScore exhibited a promising predictive ability. It may be able to serve as a visualized tool for guiding clinical decisions and selecting effective individualized treatments.

## 1 Introduction

Renal cell carcinoma (RCC) is a common urological tumor, with the sixth highest incidence among male cancer. It is estimated that 73,750 new cases and 14,830 deaths due to RCC will occur in the United States in 2020.^[1]^ There are various histological subtypes of RCC, among which clear cell renal cell carcinoma (ccRCC) accounts for approximately 70%.^[2]^ The etiology of RCC is still unclear, although heredity, smoking, obesity, and hypertension are recognized risk factors.^[3–5]^ Patients with ccRCC lack symptoms in the early stage, so it is usually found incidentally on imaging. When typical symptoms such as hematuria, lumbago, and abdominal mass take place, many patients are diagnosed with metastatic carcinoma, whose 5-year overall survival (OS) rate is dismal at 8% to 12%.^[6]^ In addition, 20% to 30% localized ccRCC patients will progress to metastasis despite nephrectomy.^[7]^ Thus, there is an exigence for new molecular markers to identify patients with a high risk of progress and poor prognosis to alert clinicians.

Hypoxia is a characteristic of solid tumors that profoundly affects the expression of many non-coding RNAs.^[8,9]^ It is reported that hypoxia directly contributes to many hallmarks of cancer, including reprogramming metabolism, proliferation, invasion and metastasis, apoptosis, and resistance to therapy.^[10,11]^ Hypoxia inducible factor (HIF) is a protein complex, composed of either HIF-1a or HIF-2a and HIF-1b/ARNT subunits, that centrally regulates cellular oxygen detection and adaptation.^[12]^ Furthermore, HIF-1a activity is commonly diminished by chromosomal deletion in ccRCCs, and increased HIF-1 activity reduces tumor burden in xenograft tumor models. Conversely, polymorphisms at the HIF-2a gene locus pre-dispose to the development of ccRCCs, and HIF-2a promotes tumor growth. Many studies have uncovered the critical roles of hypoxia in the tumor microenvironment, including cell proliferation and differentiation and tumor angiogenesis and immune infiltration. Hypoxia can activate the hypoxia-inducible factors and then induce adaptive changes within a cancer cell, which results in tumor progression and treatment resistance.^[13]^ Hypoxia and overexpression of HIF are associated with prognosis in various cancers. For example, in 1 study a poor disease outcome group had significantly higher HIF-1α expression in stage IIB–IIIB cervical cancer.^[14]^ It is found that the HIF family of proteins (either directly or combined with glucose uptake and the glycolysis pathway) regulates many downstream targets to contribute to the development and progression of ccRCC.^[15]^

Some studies have reported that hypoxia-related genes may affect the prognosis of typical cancer patients, such as gastric cancer.^[16]^ Furthermore, previous studies have explored the close relations between the hypoxia and several cancers, including glioblastoma, colorectal cancer, breast cancer, and hepatocellular carcinoma.^[17–21]^ Chen et al^[22]^ identified a hypoxia-associated long non-coding RNA signature and established a nomogram predicting prognosis of ccRCC, which demonstrated the potential of hypoxia factor in ccRCC. However, whether hypoxia-related genes can be used as a prognostic indicator of ccRCC and the molecular mechanism of these hypoxia-related genes in ccRCC is still a mystery. In the past few years, the role of hypoxic microenvironment in tumors has always been a hot point, but there have been few reports on the relationship of hypoxia genes and ccRCC. This study will explore the relationship between hypoxia genes and the prognosis of ccRCC by using bioinformatics analysis, hoping to lay the foundation for further research on the molecular mechanism of hypoxia genes in ccRCC.

Bioinformatics is a new inter-disciplinary subject combining molecular biology and information technology. In recent years, the use of big data for bioinformatics analysis is gradually being applied to various tumor fields. Bioinformatics analysis can help us focus on specific molecules from the expression data, such as RNA-seq data, thereby helping to reveal the molecular mechanism of disease.^[23]^ In this study, we obtained and processed RNA transcriptome data and clinical information of ccRCC patients from the Cancer Genome Atlas (TCGA) database and the International Cancer Genome Consortium (ICGC) database. We then further established a hypoxia-related multigene prognosis model and visualized an optimized nomogram convenient for clinicians to use, which was verified using ICGC data.

## 2 Methods

### 2.1 Data collection and processing

The hallmark gene sets of hypoxia, including 200 genes, were downloaded from the Molecular Signatures Database (MSigDB version 6.0; http://software.broadinstitute.org/gsea/msigdb/index.jsp). The original RNA seq dataset and clinical information of the ccRCC dataset were downloaded from the TCGA database (https://www.cancer.gov/tcga) and ICGC database (https://icgc.org/). For TCGA and ICGC cohort, we removed patients if their clinical information and follow-up data were incomplete, only patients with transcriptome data and complete clinical information could meet the inclusion criteria. R software (version 4.0.0; https://www.r-project.org/) was used to standardize and process data.

### 2.2 Screening and visualization of hypoxia-related differentially expressed genes

We used the “limma” R package to screen the RNA raw data and excluded genes whose average count value was lower than 1. All hypoxia genes whose |log2 fold change| was above 1 and false discovery rate was lower than 0.05 were defined as hypoxia-related differentially expressed genes (DEGs). The “pheatmap” algorithm was used to draw a volcano plot and a heatmap for the hypoxia-related DEGs.

### 2.3 Functional enrichment and protein–protein interaction network analysis

We evaluated the hypoxia-related DEGs according to their Gene Ontology (GO), including analysis of cellular components, molecular function, and biological process (BP) involvement, and also based on the Kyoto Encyclopedia of Genes and Genomes (KEGG) pathway enrichment analysis. Both enrichment analyses were performed using “clusterProfiler,” “org.Hs.eg.db,” “enrichplot,” “ggplot2”, and “GOplot” packages in R software. *P* < .05 and false discovery rate < 0.05 were considered statistically significant. Then, we used the string database (https://string-db.org/) to construct the protein–protein interaction (PPI) network of hypoxia-related DEGs (CombinedScore = 0.40) and imported the data into Cytoscape software (version 3.7.2; https://cytoscape.org/) to visualize the interaction of the PPI network.

### 2.4 Construction of prognostic model

Among a total of 537 ccRCC patients, data related to 7 patients were excluded from analysis because they were missing some clinical information. Using the “survival” R package, we performed univariate Cox regression analysis on all hypoxia-related DEGs to narrow the candidate list to 16 hub hypoxia genes related to ccRCC prognosis (*P* < .001). We also evaluated the impact of these hypoxia genes on the survival time and outcome of patients through multivariate Cox proportional hazard regression. Next, we constructed a predictive model using expression data of the 6 hub hypoxia genes. The risk score of each patient was calculated according to the following equation:

$$\begin{array}{l} Risk\;score=\beta 1\times ExpGene1+\beta 2\times ExpGene2+\beta 3\times ExpGene3+\beta 4\\ \;\;\;\;\;\;\times ExpGene4+\beta 5\times ExpGene5+\beta 6\times ExpGene6, \end{array}$$

where β represents a coefficient value that was identified based on the impact of each gene, and Exp represents the expression level of each hypoxia gene. The patients were divided into 2 subgroups (low-risk and high-risk) using the median risk score as a threshold. OS of the 2 groups was evaluated by the Kaplan-Meier plot and log-Rank test using “survival” and “survminer” packages in R. The plots of the survival status of patients and heatmap were drawn using the “pheatmap” package in R. Additionally, we carried out receiver operating characteristic (ROC) curve analysis using the “survivalROC” package to evaluate the overall diagnostic performance of the proposed model. Moreover, we performed univariate Cox regression analysis and multivariate Cox proportional hazards regression with the “survival” package to investigate whether the risk score was an independent prognosis predictor of ccRCC. RiskScore, age, gender, tumor subtype, pathological stage, and histological grade were used as covariates.

### 2.5 Correlation between the expression of the 6 hypoxia-related genes and survival time as well as clinical characteristics of ccRCC patients

We obtained the relationship between the expression levels of these 6 hypoxia-related genes and the survival time of ccRCC patients based on the Gene Expression Profiling Interactive Analysis database (GEPIA; http://gepia.cancer-pku.cn/). After that, the analysis of the 6 genes combined with clinical characteristics was performed.

### 2.6 The establishment and validation of nomogram

Age, gender, stage, grade, T, N, M, and RiskScore were used to construct the nomogram, using the“rms” and “survival” packages to ease the use of this new prognostic tool and allow quantitative prognostic evaluation of patients. We refer to this optimized nomogram as the new model and the corresponding nomogram without the RiskScore variable as the old model. Then, C-index, calibration curves, decision curve analysis (DCA), integrated discrimination improvement (IDI), and net reclassification index (NRI), which were used as indices to evaluate the advantages and disadvantages of the new and old models, were calculated and drawn to determine which model is better. After that, C-index and calibration curves were calculated and drawn to assess the consistency between actual and predicted survival between TCGA-ccRCC and ICGC-ccRCC cohorts, respectively.

## 3 Results

### 3.1 Differentially expressed hypoxia-related genes

**Figure 1:**
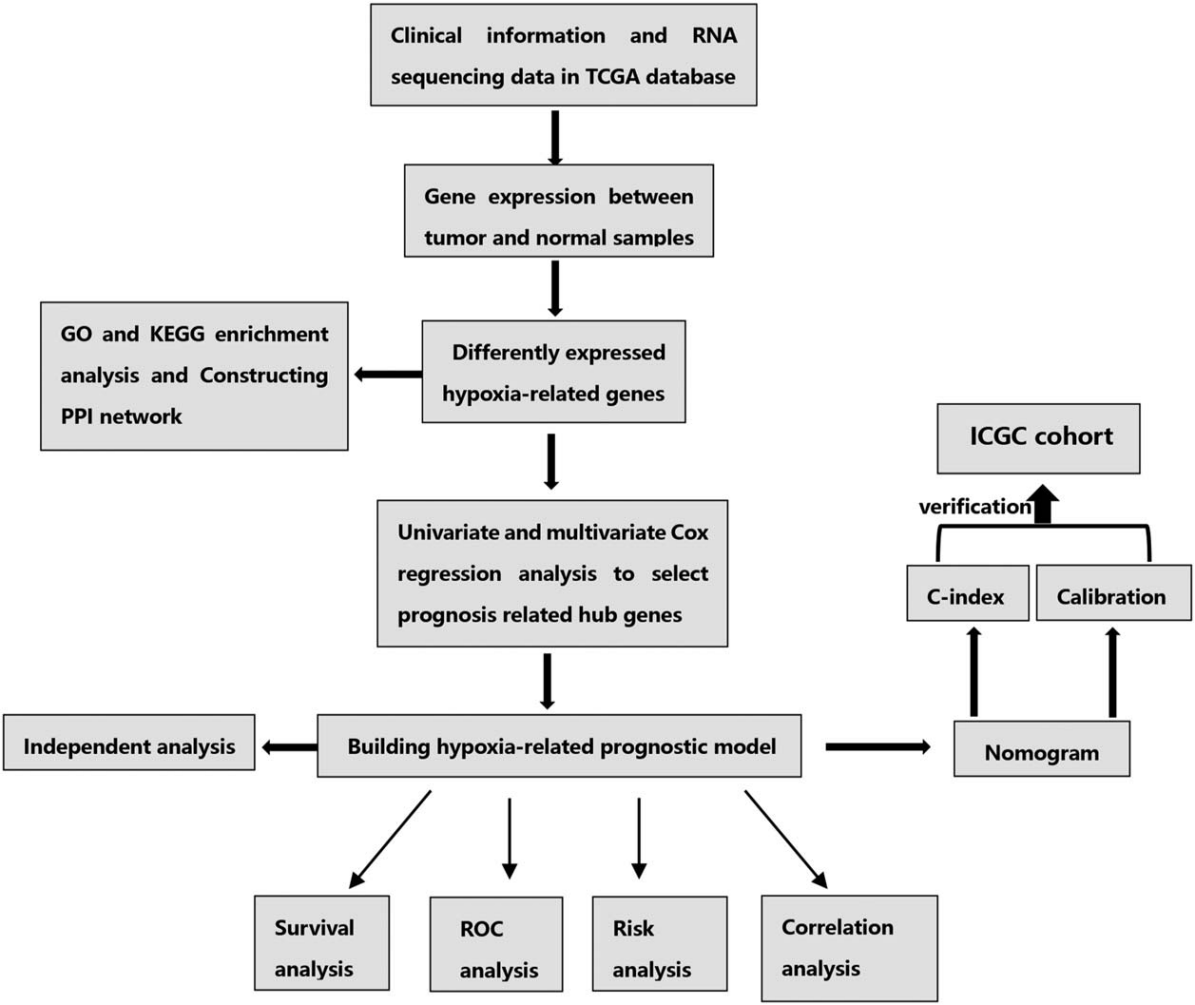
The workflow of this study. GO = Gene Ontology, ICGC = International Cancer Genome Consortium, KEGG = Kyoto Encyclopedia of Genes and Genomes, PPI = protein–protein interaction, ROC = receiver operating characteristic, TCGA = the Cancer Genome Atlas.

**Figure 2:**
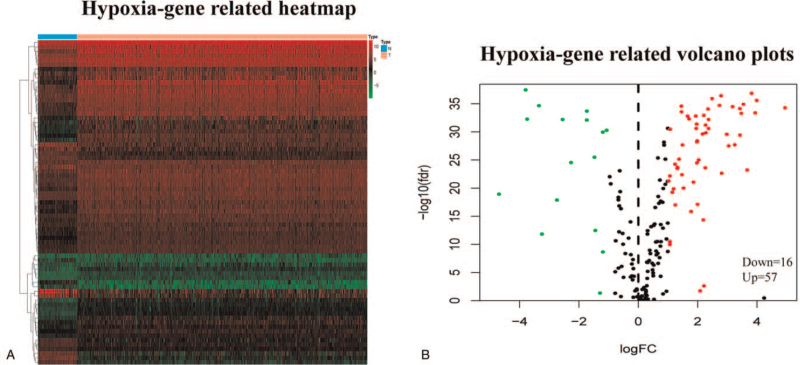
The differentially expressed hypoxia genes in clear cell renal cell carcinoma (ccRCC). (A) Hypoxia-gene related heatmap. (B) Hypoxia-gene related volcano plots.

In this study, we used several advanced algorithms to identify hypoxia-related DEGs. Figure 1 shows the workflow of our research. Finally, a total of 537 and 91 patients from the TCGA (normal samples: 72) and ICGC databases (normal samples: 45), respectively, were included in the next study. The characteristics of ccRCC patients from TCGA and ICGC databases are shown in Table 1. We identified 57 upregulated and 16 downregulated hypoxia genes that were eligible for further screening via the “limma” package in R. The identified hypoxia-related DEGs are shown in heatmap and volcano plots in Figure 2A and B.

**Table 1:** Characteristics of patients from TCGA and ICGC databases.

Characteristic	TCGA (%)	ICGC (%)
AJCC stage		
I	269 (50.1)	48 (52.7)
II	57 (10.6)	12 (13.2)
III	125 (23.3)	13 (14.3)
IV	83 (15.5)	9 (9.9)
Not available	3 (0.6)	9 (9.9)
Grade		
I	14 (2.6)	13 (14.3)
II	230 (42.8)	48 (52.7)
III	207 (38.5)	15 (16.5)
IV	78 (14.5)	14 (15.4)
Not available	8 (1.5)	1 (1.1)
Sex		
Male	346 (64.4)	52 (57.1)
Female	191 (35.6)	39 (42.9)
Age (mean ± standard deviation)	60.59 ± 12.14	60.47 ± 10.03
Tumor samples	537	91
Normal tissue samples	72	45

AJCC = American Joint Committee on Cancer, ICGC = International Cancer Genome Consortium, TCGA = the Cancer Genome Atlas.

### 3.2 Enrichment analysis and construction of the PPI network

**Figure 3:**
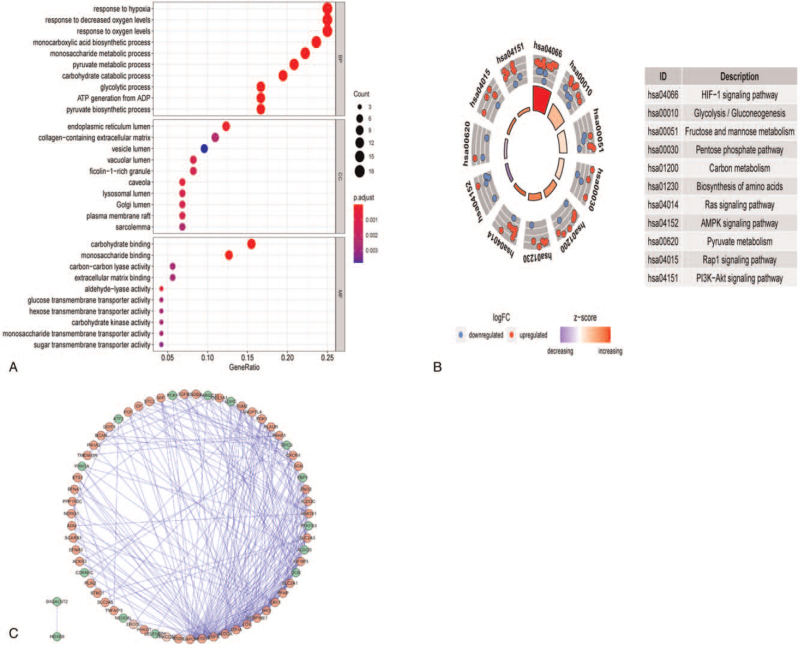
Enrichment analysis and protein–protein interaction (PPI) network. (A) GO analysis of differentially expressed hypoxia genes. (B) KEGG analysis of differentially expressed hypoxia genes. (C) PPI network of differentially expressed hypoxia genes. Green circles: down-regulation; red circles: up-regulation. BP = biological process, CC = cellular component, GO = Gene Ontology, KEGG = Kyoto Encyclopedia of Genes and Genomes, MF = molecular function.

Next, we performed GO and KEGG enrichment analysis. The GO analysis results showed that hypoxia-related DEGs can be enriched in several basic BPs, including “response to hypoxia,” “response to decreased oxygen levels,” “response to oxygen levels,” and “monosaccharide metabolic process” (Fig. 3A). The KEGG pathway analysis results showed that the identified hypoxia-related DEGs were involved in the “HIF−1 signaling pathway,” “Glycolysis/Gluconeogenesis,” “Ras signaling pathway,” and “AMPK signaling pathway” (Fig. 3B). The PPI network, which included hypoxia-related DEGs, consisted of 65 nodes and 254 edges, as shown in Figure 3C. The above results indicate that these genes may play an important role in the occurrence and development of ccRCC through these signaling pathways, and there are abundant protein interactions between them.

### 3.3 Identification of prognostic hypoxia-related genes

**Figure 4:**
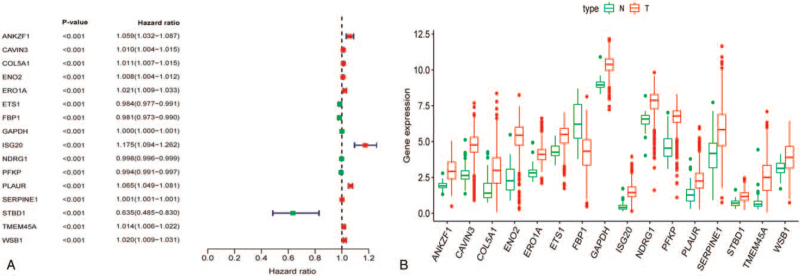
Univariate Cox regression analysis for identification of prognostic hypoxia genes. (A) Forest map of hypoxia genes related to ccRCC survival via univariate Cox regression. (B) Boxplot of hypoxia genes associated with ccRCC survival. ccRCC = clear cell renal cell carcinoma, N = normal, T = tumor.

Based on the 73 important hypoxia-related DEGs, we conducted univariate Cox regression analysis to investigate the prognostic value of these genes. The analysis revealed 16 candidate genes potentially associated with ccRCC prognosis (Fig. 4A and B; *P* < .001). Next, we evaluated the association between these identified prognostic-associated candidate genes and patient survival time and clinical outcomes through multiple stepwise Cox regression. This analysis revealed 6 hub hypoxia genes (ANKZF1, ETS1, FBP1, PFKP, PLAUR, and SERPINE1) that showed potential as independent predictors for ccRCC prognosis (Table 2).

**Table 2:** Prognostic-related genes via the multivariate Cox regression analysis.

Gene symbol	Coefficient	HR	*P* value
ANKZF1	0.054262	1.055762	4.24E-05
ETS1	−0.00974	0.990305	.009003
FBP1	−0.01052	0.989535	.016046
PFKP	−0.00426	0.995746	.012756
PLAUR	0.048648	1.04985	1.41E-07
SERPINE1	0.000519	1.000519	.048343

### 3.4 Construction of hypoxia-based prognostic model

Next, we constructed a hypoxia-based model using the 6 identified hub genes. The following formula was calculated to generate the RiskScore for each patient:

$$\begin{array}{l} Risk\;score=0.054262\times \text{ANKZF}1+\left(-0.00974\times \text{ETS}1\right)+0.048648\\ \;\;\;\times \text{PLAUR}+0.000519\times \text{SERPINE}1+\left(-0.00426\times \text{PFKP}\right)+\left(-0.01052\times \text{FBP}1\right) \end{array}$$

**Figure 5:**
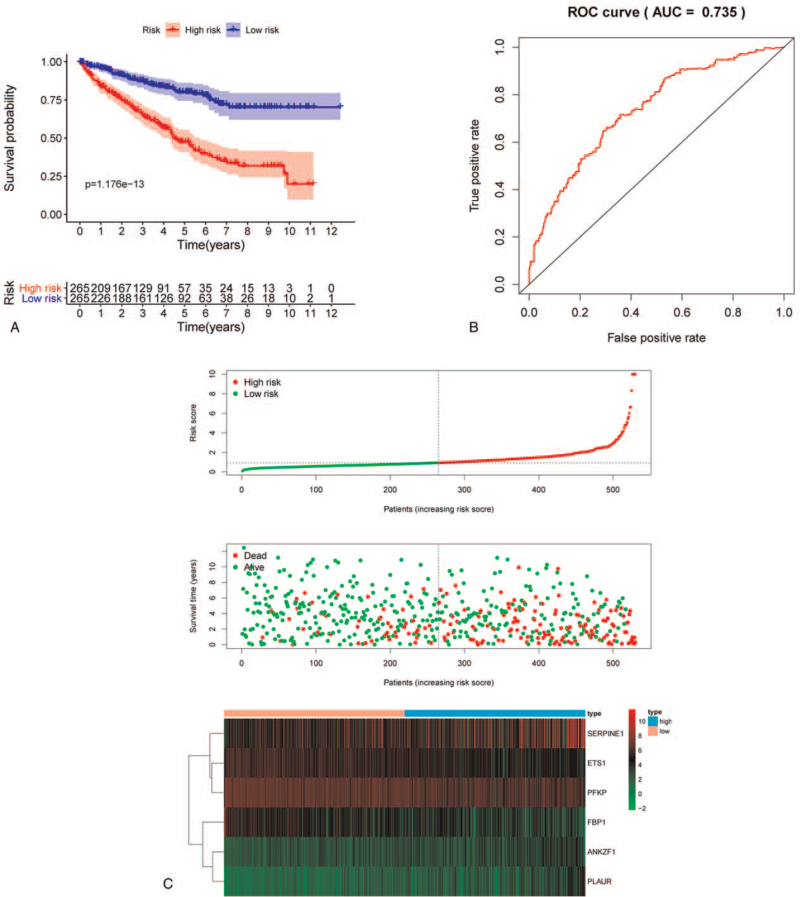
RiskScore analysis of prognostic model based on 6 hypoxia-related genes. (A) Survival curves for low- and high-risk subgroups. (B) ROC curves for forecasting 3- and 5-year overall survival based on RiskScore. (C) Expression heat map, RiskScore distribution, and survival status. ROC = receiver operating characteristic.

To further evaluate the predictive potential of the proposed predictive model, we evaluated the survival outcome of patients with ccRCC according to their calculated RiskScore (Fig. 5A). We calculate the average value of the total RiskScore, and use this value as the threshold to divide patients into high- and low-risk groups. The RiskScore of the high-risk group is greater than the average, while the low-risk group is the opposite. The results showed that patients with high RiskScores had poor OS, whereas patients with low RiskScores had longer OS. Furthermore, a time-dependent ROC curve analysis revealed that the AUC value, which predicted 3-year and 5-year OS, was 0.718 and 0.742, respectively (Fig. 5B), suggesting that the proposed prognostic model had good ability to predict the long-term outcome of ccRCC patients. Figure 5C shows the expression heatmap, patient survival status, and risk scores of the 6 hypoxia-related signatures in the low- and high-risk patient subgroups. These results indicate that the hypoxia-related model has a good prediction ability and distinguishing ability.

### 3.5 Independent analysis of the hypoxia-related model

**Figure 6:**
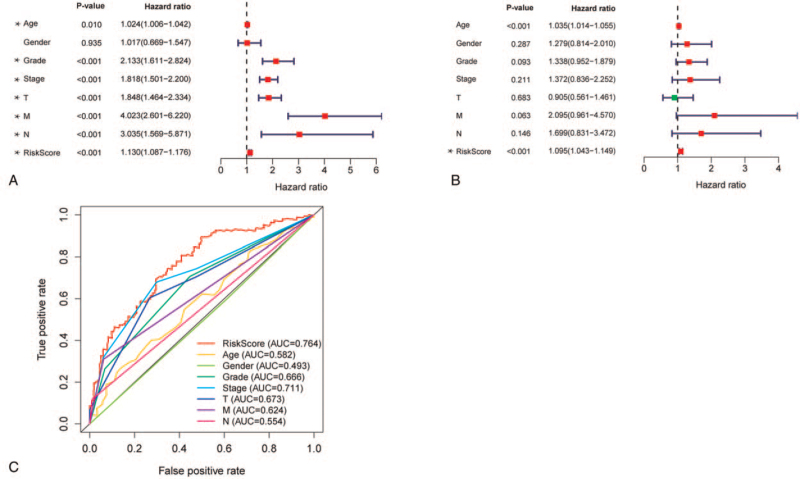
Hypoxia-related gene signatures are significantly associated with ccRCC survival. (A) Forest plot of associations between risk factors and the survival of ccRCC via univariate Cox regression analysis. (B) The hypoxia-associated gene signature is an independent predictor of ccRCC via multiple Cox regression analysis. (C) Multiindex ROC curve of risk score and other indicators. ccRCC = clear cell renal cell carcinoma, ROC = receiver operating characteristic.

To explore whether the RiskScore is independent of other clinical characteristics such as staging, we conducted an independence test for this model. The prognostic values of different clinical characteristics of ccRCC patients were also evaluated by univariate Cox regression analysis, which revealed that age, cancer grade, stage, TNM, and RiskScore were related to OS (Fig. 6A, *P* < .05). Moreover, multiple regression analysis showed that prognostic factors such as age and RiskScore could independently predict OS (Fig. 6B, *P* < .001). Overall, the RiskScore was a robust predictor that was independent of the staging and grading of the patients. In addition, the RiskScore's accuracy (AUC = 0.764) is better than the staging and grading system in the multiindex ROC curve (Fig. 6C).

### 3.6 Verification of correlation between the expression of the 6 hypoxia-related genes and survival time as well as clinical characteristics of ccRCC patients

**Figure 7:**
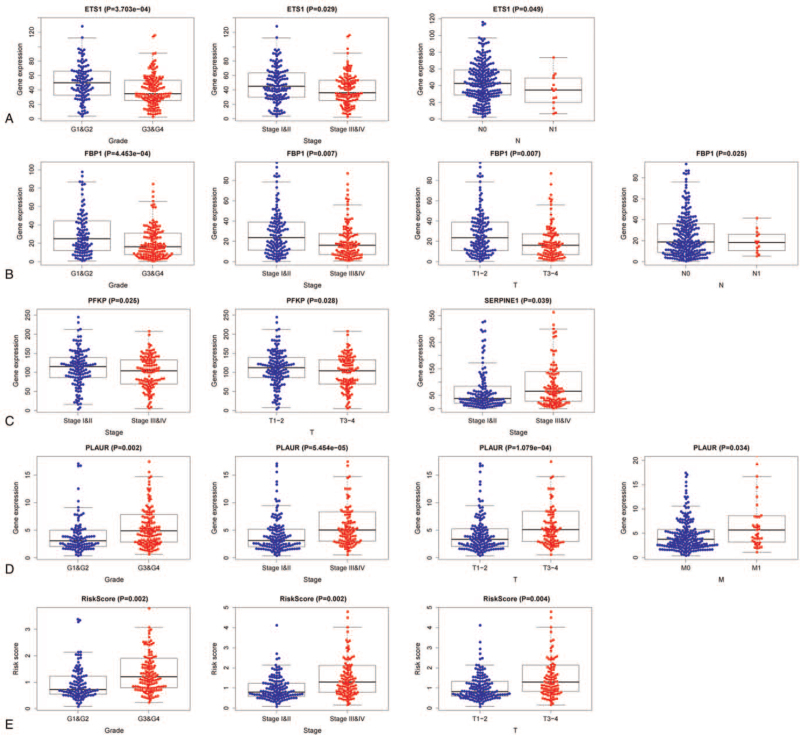
Relationships of the hypoxia genes in the model with the clinical characteristics of patients.

**Figure 8:**
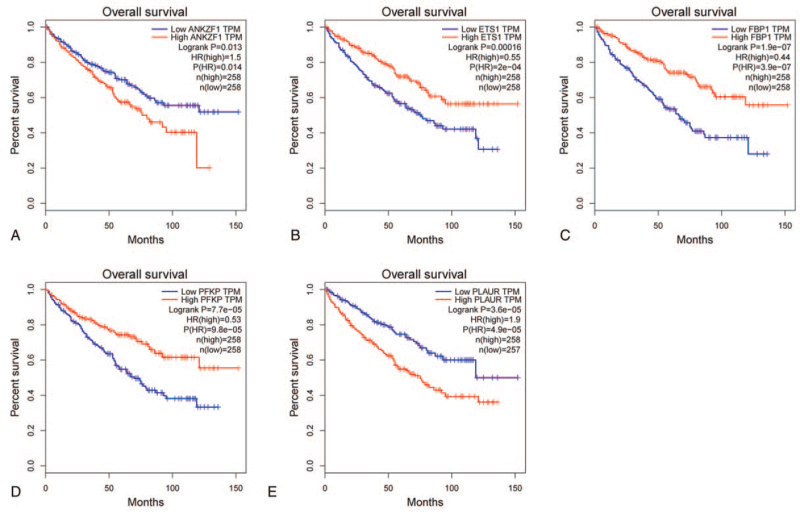
Validation the prognostic value of hub hypoxia genes in ccRCC by GEPIA online tool. ccRCC = clear cell renal cell carcinoma, GEPIA = Gene Expression Profiling Interactive Analysis.

We investigated the correlation between the 6 hypoxia-related genes and clinical characteristics, and the results revealed that these genes were almost related to the patient's staging and grading (Fig. 7A–E). We also evaluated the correlation between the expression of the 6 hypoxia-related genes and survival time via GEPIA online tools, which revealed that the expression levels of ANKZF1, ETS1, FBP1, PFKP, and PLAUR had a positive or negative correlation with the ccRCC patient's OS (Fig. 8A–E). The above results indicated that the expression of these 6 genes had a robust correlation with the prognosis of ccRCC patients and the 6 hypoxia-related genes might be suitable for predicting the prognosis of ccRCC patients.

### 3.7 Validation of the nomogram

**Figure 9:**
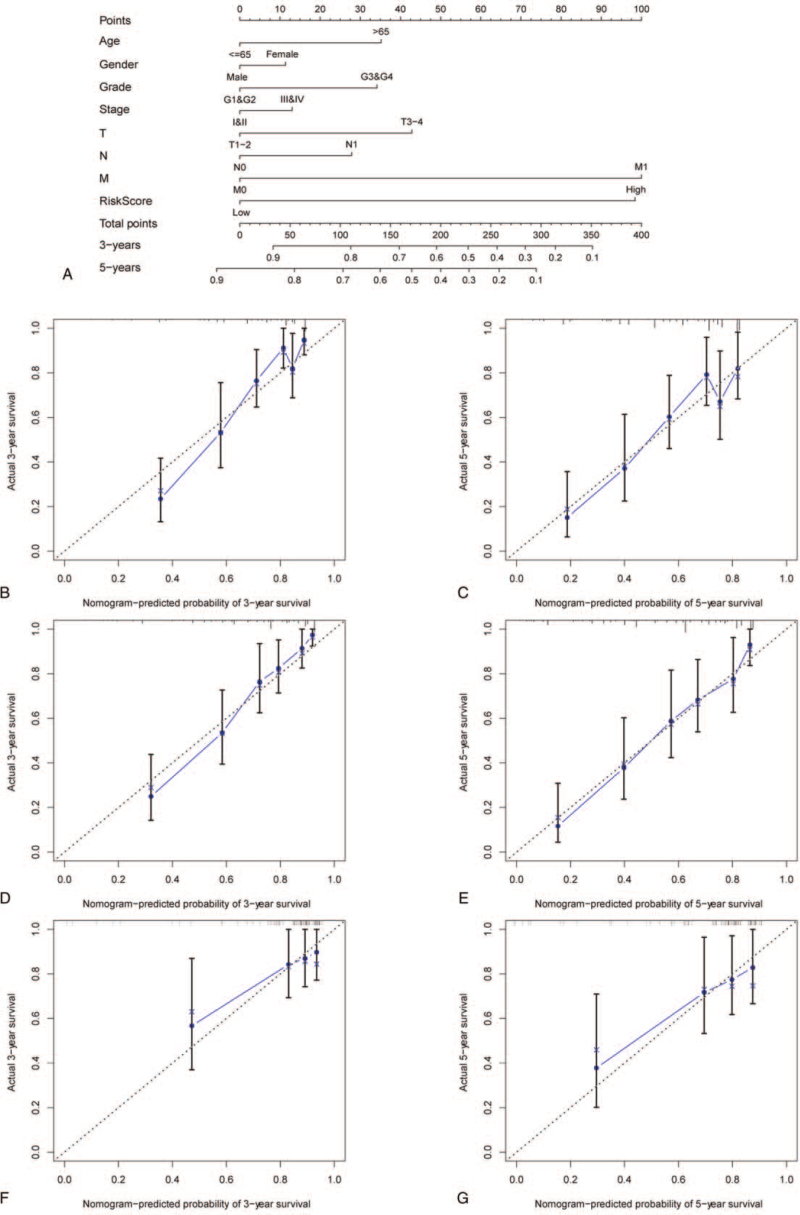
The nomogram can predict the prognosis probability in ccRCC. (A) A nomogram of the TCGA cohort used to predict the OS. (B, C) Calibration maps used to predict the (B) 3-year and (C) 5-year survival in the old model. (D, E) Calibration plots for (D) 3-year and (E) 5-year survival in the new model (training set). (F, G) Calibration plots for (F) 3-year and (G) 5-year survival in the ICGC cohort (test set). The x-axis and y-axis represent the predicted and actual survival rates of the nomogram, respectively. The solid line represents the predicted nomogram, and the vertical line represents the 95% confidence interval. ccRCC = clear cell renal cell carcinoma, ICGC = International Cancer Genome Consortium, OS = overall survival, TCGA = the Cancer Genome Atlas.

**Figure 10:**
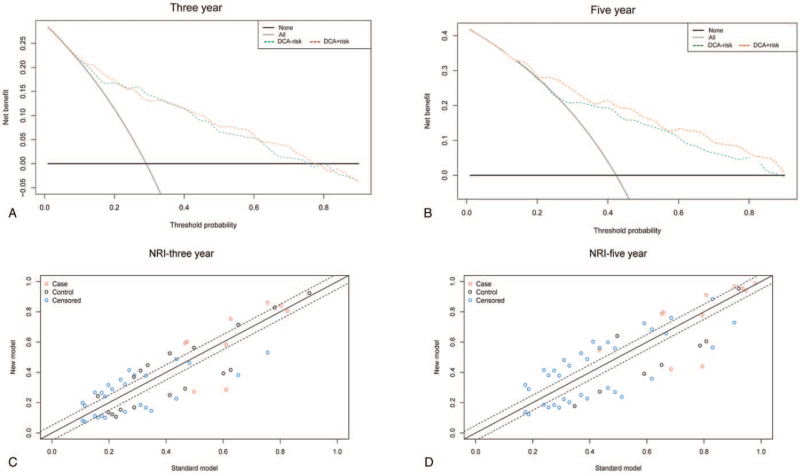
Comparative analysis of new and old models. (A) DCA curve of 3-year survival rate of ccRCC patients. DCA-risk stands for “old model”; DCA + risk stands for “new model.” (B) DCA curve of 5-year survival rate of ccRCC patients. (C) NRI plot of 3-year survival rate of ccRCC patients. (D) NRI plot of 5-year survival rate of ccRCC patients. ccRCC = clear cell renal cell carcinoma, DCA = decision curve analysis, NRI = net reclassification index.

We also designed a nomogram for ccRCC prognosis based on the signatures of the 6 hypoxia-related genes (Fig. 9A). The point of each variable could be found in a horizontal line, which was then summed and normalized into a distribution of 0 to 100 for each patient. The nomogram-predicted probability of 3- and 5-year OS for ccRCC patients could be obtained after calculating the total nomogram score. The C-index of the new model and old model was 0.773 (95% CI: 0.724–0.822) and 0.758 (95% CI: 0.705–0.811), respectively. IDI results showed that the new model was 3.7% better than the old model (*P* < .01). Calibration curve revealed that the new model was better than the old model, as shown in Figure 9B to E. After we concluded that the new model was better than the old model, we conducted external verification of the new model. The 3-year and 5-year calibration curves of the ICGC dataset are shown in Figure 9F and G. The indication of the calibration curve matches well. The C-index of the ICGC dataset was 0.710 (95% CI: 0.592–0.828). The results also showed that the new model has a better ability to predict the survival rate of ccRCC patients. In addition, DCA and NRI plots also revealed that the new model was better than the old model, as shown in Figure 10A to D.

## 4 Discussion

Since the 1990s, hypoxia-induced pathways have received great attention from researchers. Rapidly-proliferating tumor cells, incremental oxygen consumption, and structurally and functionally abnormal vasculature inside tumor tissue co-formed a unique hypoxia micro-environment of solid tumors, distinguishing cancer from normal tissue and also partly contributing to a poor clinical outcome.^[24]^ Interestingly, the hypoxia signaling pathway can sometimes also be activated by genetic alterations in cancers; for example, the dysfunction of VHL can be observed in ccRCC.^[25]^ Most ccRCC are associated with deregulation of hypoxia pathways.^[26]^ This shows that the hypoxia pathway plays an important role in ccRCC.

Increasing evidence demonstrates that there are direct or indirect interactions between the hypoxia signaling pathway and tumorigenesis or tumor progression. However, adaptive responses triggered by hypoxia containing hundreds of related proteins and genes can be both diverse and specific. Besides, intersecting with other signaling mechanisms also makes the hypoxia signaling pathway intricate and obscure. It is impractical to figure out all the steps involved in the hypoxia signaling pathway relying on present theory. Hypoxia-induced up-transcription of genes has shown a demonstrable influence on a series of tumor BPs, including cell uncontrollable proliferation and immortalization,^[27]^ angiogenesis,^[27]^ glucose metabolism,^[28]^ immune escape,^[29]^ tumor invasion and metastasis,^[30]^ and radiation resistance.^[31]^ This reveals that the differential expression of hypoxia-related genes might be a risk feature for survival of ccRCC patients. Accordingly, we focused our attention to the hypoxia-related genes of the hypoxia signaling pathway and investigate the role of these hypoxia genes in the occurrence and development of ccRCC.

Here, we tried to investigate the correlation between the survival of patients with ccRCC and hypoxia-related genes and subsequently identify genes significantly contributing to clinical outcome. After univariate Cox regression analysis and multivariate Cox regression analysis, the signature of 6 genes (ETS1, ANKZF1, PLAUR, SERPINE1, PFKP, and FBP1) were selected. We further established the multigene prognostic model of ccRCC based on the 6 genes, which was more reliable than single gene prognostic model. According to the median RiskScore, patients were divided into high-risk group and low-risk group. Subsequently, a series of analysis including ROC curve, risk plot, and survival analysis were conducted to evaluate the prognostic value of RiskScore, resultantly suggesting that RiskScore had ideal overall diagnosis and prognosis performance. In addition, results of univariate Cox regression analysis and multivariate Cox proportional hazards regression analyses revealed that RiskScore based on these hypoxia-related genes was an independent risk factor beyond TNM staging and grading. A multiple risk factors ROC curve also suggested that RiskScore was an excellent independent prognosis predictor for the 5-year survival rate of patients compared with other risk factors such as TNM staging, grading, and age. Gene signature is often applied to forecast the prognosis of a variety of tumors in the past few years,^[32]^ which is even better than TNM staging and histopathological diagnosis in some extent.^[33]^ Therefore, we believe that the model we constructed has certain value in predicting the prognosis of ccRCC patients.

Differential expression of the abovementioned 6 gene signatures and their impacts in cancer have been reported previously. Over-expression of ETS1 (E26 transformation specific-1), which belongs to the large family of transcription factors with ETS domain, was identified in a variety of solid tumors, including breast cancer, lung cancer, and specifically, renal carcinoma.^[34]^ ETS1 was also considered as an oncogene that was involved in many BPs, such as invasion, proliferation, cell metabolism, and angiogenesis, and thus was linked to unfavorable survival. For instance, Gao et al^[35]^ research shows that β6 integrin upregulates MMP3 and 9 (metalloproteinase-3 and 9) via ERK-ETS1 pathway, and subsequent the invasion of colon cancer cells. Singh et al^[36]^ research shows that EST1 can stimulate the proliferation of the hepatoma cell line Huh7 by upregulating cyclin E and CDK2 (cyclin-dependent kinase 2). Interestingly, study by Verschoor et al^[37]^ shows that ovarian cancer cells that over expressed EST1 are glycolytic reliance, suggesting that EST1 is also involved in the regulation of cancer cells energy metabolism. Additionally, EST1 expression has also been found to promote tumor angiogenesis in many cancers.^[34]^ Further research provided evidence that ETS1 played a part through regulating the expression of HIF-related genes, consistent with the results of GO and KEGG enrichment analyses in our research.^[38]^ However, our research came to the conflicting conclusion that the elevated expression of ETS1 was a protective feature for prognosis. Similar experimental conclusions have also been reported for colon cancer cells and invasive breast cancer.^[39,40]^ This may be related to the heterogeneity of the tumor. The apoptosis-inducing activity of ETS1 may also account for that. Pro-apoptotic genes, including CDKN1A (encoding p21 protein), CDKN1B (encoding p27 protein) and caspase I,^[41]^ and tumor suppressor protein genes, including p16INK4A,^[42]^ were validated as targets of ETS1.^[43]^ ANKZF1 is a cofactor that binds to p97 and regulates its cellular biological functions, such as protein quality control, apoptosis, autophagy, DNA damage repair, and transcriptional activation.^[44]^ The research about ANKZF1 has been reported in recent years, some of which suggest that it may be associated with tumor angiogenesis.^[45]^ Plasminogen activator urokinase receptor (PLAUR) acts as a receptor for urokinase plasminogen activator and plays a role in localizing and promoting plasmin formation. In the mechanisms of tumorigenesis, PLAUR can promote tumor invasion by remodeling of the extracellular matrix and tumor microenvironment and actively promotes DNA repair in cancer cells.^[46]^ In addition, PLAUR is highly expressed in most solid cancers and serves as a marker of poor prognosis, consistent with the results of GEPIA database in our research. Wu et al^[47]^ research showed that Serpin Family E Member 1 (SERPINE1) was high-expressed in glioma and up-regulation of miR-1275 activated p53 signaling pathway via regulating SERPINE1 and therefore suppressed glioma cell proliferation, invasion, and migration, whereas promoted cell apoptosis. Some study also showed that overexpression of SERPINE1 enhances tumor cell migration and invasion and plays a key role in metastasis development, conferring poor prognosis.^[48]^ Up-regulation of phosphofructokinase-platelet (PFKP), one of the isoforms of phosphofructokinase-1 (PFK-1), had been frequently reported in different types of cancer. Shen et al^[49]^ detected the expression of PFKP in lung cancer and put forward that the overexpression of PFKP played a crucial role in tumor initiation and progression in lung cancer by glycolysis. Our study also found that PFKP was enriched in the glycolysis pathway according to the results of KEGG analysis. This indicates that FPKP may also affect the occurrence and development of ccRCC through glycolysis. After detecting the expression of PFKP in human glioblastoma and investigating a possible regulation mechanism, Lee et al^[50]^ drew a conclusion that decreased degradation of PFKP due to phosphorylation correlated with clinical aggressiveness of glioblastoma. Li et al^[51]^ research revealed that FBP1 knockdown in prostate cancer could activate autophagy mediated by the AMPK-mTOR signaling pathway, while inhibition of the AMPK-mTOR signaling pathway could reverse FBP1 knockdown-mediated autophagy and apoptosis. Our KEGG enrichment analysis results also indicate that AMPK signaling pathway may play an important role in ccRCC (*P* < .05). However, whether FBP1 can also influence the occurrence and development of ccRCC through AMPK signaling pathway is worthy of further study. Another research showed that FBP1, which was the crucial enzyme in gluconeogenesis, was general absence in ccRCC.^[51]^ They provided an opinion and relevant evidence that FBP1 worked as an opposer by antagonizing both HIF mediated hypoxia adaption responses and glycolytic flux within tumor tissue in ccRCC progression.^[51,52]^ However, further clinical trials are needed to validate our observations and the mechanisms underlying the prognostic value of these genes in ccRCC also deserve further experimental exploration.

In addition, the 6 hypoxia-related genes chosen for RiskScore calculation seemed to be expressed more in high staging tumors, whereas others were expressed more in low staging tumors. Whether these genes are up-regulated or down-regulated in tumor tissues, the association between these genes and the tumor clinical grades and TNM stages could be observed in the present study, indicating that multiple prognostic hypoxia-related genes might co-effect in the progression of ccRCC.

A novel and robust prognostic tool for patients with ccRCC has been in high demand. Hence, by combining clinical-pathological features and prognostic gene signatures, a nomogram was established to predict individual survival probability. Different from previous prognostic systems, either comprehensive or simply based on clinicopathologic characteristics and the current TNM staging method, ideas about the prognostic implications of hypoxia-related genes are the focus of this study. Necessarily, we compared the optimized prognosis nomogram and the previous one with several evaluations, including C-index, calibration curves, NRI, IDI, and especially, DCA, which was more practical than the typical ROC curve analysis since it took the consequences of clinical strategies into consideration.^[53]^ The optimized nomogram was clearly the superior one, with better accuracy and discrimination. Unsurprisingly, subsequent external validation in the ICGC database showed the promising performance of the optimized nomogram. The above results indicate that a novel prognosis nomogram optimized via RiskScore, a new independent prognosis predictor, is feasible. We believe that the findings of our research could provide robust prognostic indicators and underlying therapeutic targets for patients with ccRCC, and more importantly, provide insight into the correlation between hypoxia-related gene expression and clinical outcomes. We believe that patients may benefit from our study.

Although we made several accomplishments in the investigation of novel prognostic biomarkers, there were several limitations in this study. Firstly, our study was designed on the basis of a retrospective analysis and prospective research should be performed to verify the outcomes. Secondly, because of the limited clinical information, some other key clinical pathological features, such as lymph node invasion, are not included in the nomogram. Finally, we did not further investigate and verify the roles that these hypoxia-related genes played in tumorigenesis and tumor progression in ccRCC through experimental research. Further functional studies of these genes identified will be necessary in the future.

## 5 Conclusions

Our study reveals that the differential expression of hypoxia-related genes is associated with the OS of patients with ccRCC. We further established the prognostic model of ccRCC based on these genes identified, which showed a good predictive and discerning ability. And the novel nomogram optimized via the hypoxia-related genes-based RiskScore exhibited promising predictive ability. It may be able to serve as a prognostic tool for guiding clinical decisions and developing effective individualized treatment.

## Acknowledgments

The results of this study are based on data from TCGA (https://www.cancer.gov/tcga) and ICGC (https://icgc.org/). We thank the authors who provided the data for this study. We thank LetPub (www.letpub.com) for its linguistic assistance during the preparation of this manuscript.

## Author contributions

Bo Zhang designed the experiments. Zhengtian Li, Chan Li, Jun Huang, Zhenpei Wen, Hening Li, and Wenkang Yang performed research. Zhengtian Li and Gang Du analyzed the data. Zhengtian Li, Rong Zhao, and Wenkang Yang wrote the manuscript. All of the authors reviewed the article.

**Data curation:** zhengtian li, Rong Zhao, Zhenpei Wen.

**Formal analysis:** zhengtian li, Gang Du, Rong Zhao, Jun Huang, Zhenpei Wen.

**Funding acquisition:** Gang Du, Wenkang Yang, Bo Zhang.

**Investigation:** Chan Li.

**Software:** zhengtian li, Chan Li, Hening Li.

**Validation:** zhengtian li, Rong Zhao, Hening Li.

**Visualization:** zhengtian li.

**Writing – original draft:** zhengtian li, Rong Zhao, Wenkang Yang, Chan Li, Jun Huang, Zhenpei Wen, Hening Li.
